# Ageing and Low-Level Chronic Inflammation: The Role of the Biological Clock

**DOI:** 10.3390/antiox11112228

**Published:** 2022-11-11

**Authors:** Barbara Colombini, Monica Dinu, Emanuele Murgo, Sofia Lotti, Roberto Tarquini, Francesco Sofi, Gianluigi Mazzoccoli

**Affiliations:** 1 Department of Experimental and Clinical Medicine, University of Florence, 50134 Florence, Italy; 2 Department of Medical Sciences, Division of Internal Medicine and Chronobiology Laboratory, Fondazione IRCCS “Casa Sollievo della Sofferenza”, Opera di Padre Pio da Pietrelcina, 71013 San Giovanni Rotondo, Italy; 3 Division of Internal Medicine I, San Giuseppe Hospital, 50053 Empoli, Italy

**Keywords:** circadian, biological clock, ageing, ER stress, UPR, inflammation, inflammasome

## Abstract

Ageing is a multifactorial physiological manifestation that occurs inexorably and gradually in all forms of life. This process is linked to the decay of homeostasis due to the progressive decrease in the reparative and regenerative capacity of tissues and organs, with reduced physiological reserve in response to stress. Ageing is closely related to oxidative damage and involves immunosenescence and tissue impairment or metabolic imbalances that trigger inflammation and inflammasome formation. One of the main ageing-related alterations is the dysregulation of the immune response, which results in chronic low-level, systemic inflammation, termed “inflammaging”. Genetic and epigenetic changes, as well as environmental factors, promote and/or modulate the mechanisms of ageing at the molecular, cellular, organ, and system levels. Most of these mechanisms are characterized by time-dependent patterns of variation driven by the biological clock. In this review, we describe the involvement of ageing-related processes with inflammation in relation to the functioning of the biological clock and the mechanisms operating this intricate interaction.

## 1. Introduction

Ageing is related to the gradual waning of the efficiency and aptitude of cells/tissues/organs to signal reparative and regenerative processes in response to internal and external stress, thereby impeding the progress of age-related diseases. This complex process is strictly linked to oxidative damage and involves immunosenescence and tissue impairment triggering inflammation and inflammasome formation. Essentially, ageing is hallmarked by low-level, systemic, chronic inflammation acknowledged as “inflammaging” [1]. Other age-related processes include augmented levels of reactive oxygen species (ROS) and endoplasmic reticulum (ER) stress-mediated unfolded protein response (UPR) [2,3] with disruption in Ca^2+^ balance and triggering of IRE1α, PERK, and ATF6 downstream signaling pathways, with inflammation and inflammasome formation [4]. The ER plays a key role in cell homeostasis as the principal cell compartment implicated in protein synthesis, folding, modification, and secretion [2,3]. The stress response prompted by disproportionate misfolded or unfolded proteins amassing in the ER lumen is termed UPR. UPR activates various signaling pathways and transcriptional processes to block protein translation, degrade misfolded proteins, and augment the assembly of chaperones required for protein folding, ultimately re-establishing ER homeostasis to avoid cell apoptosis [2,3]. A main protein turnover pathway through which cellular constituents are transported into the lysosomes for degradation and reprocessing is autophagy. This intracellular process preserves cellular homeostasis under stress conditions and its derangement could initiate physiological alterations. The activity of autophagic processes declines during ageing, resulting in the build-up of damaged macromolecules and organelles and aggravation of ageing-associated diseases [5].

The molecular processes involved in ageing are regulated by oscillatory patterns controlled by the circadian clock circuitry and mainly encompass derangement of inflammation and autophagy with immunesenescence. The complex functioning of the immune system is rhythmically ordered on different timescales, with prevalence of roughly 24-h periodicity termed circadian (from the Latin circa, approximately, and dies, within a day), showing a well-arranged time-qualified organization of the levels of humoral factors and cellular effectors with simultaneous or opposing phases of phagocytic, complement, lysozyme and peroxidase activity in innate immunity, and antibody and cytokine production, leukocyte trafficking, proliferation and apoptosis in adaptive immunity [6,7,8,9]. In the peripheral blood of healthy humans, lymphocyte subsets show 24-h rhythmic fluctuations impacting the amplitude and type of immune response, with the predominance of cytotoxic T cells during daytime and T helper cells at nighttime [10]. The temporal patterns originate from circadian variations of bone marrow production, turnover and redistribution of blood cells, as well as cell mobilization and migration to lymphatic system and peripheral tissues, implicating cyto/chemokines, hormones (cortisol, prolactin, growth hormone, thyroid stimulating hormone), sympathetic nerve fibers, and biogenic amine neurotransmitters (epinephrine, melatonin) [11,12,13,14,15].

Studies performed in innate immune system cells and peripheral blood mononuclear cells showed the presence of biological clocks in inflammatory and immune competent cells [16,17]. Furthermore, circadian rhythmicity regulates the transcriptional processes controlling the expression of genes enriching the signaling pathways involved in inflammatory processes, such as the nuclear factor κB (NF-kB) and the NLR3P3 inflammasome pathway [18]. These multifaceted interactions represent a promising target for valuable interventions to thwart the progression of physiological changes leading to a decline in the organism’s adaptive capacity and weakening of biological functions.

## 2. The Circadian Clock Circuitry

Physiology and behavior of living beings, scheduled in keeping with sleep/wake, rest/activity, and fasting/feeding cycles, show nychthemeral variations driven by the circadian timing system (CTS), a hierarchical network of biological oscillators comprising a master pacemaker in the suprachiasmatic nuclei (SCN) of anterior hypothalamus [19]. The CTS drives extra-SCN cerebral clocks and self-sustained oscillators in the peripheral tissues through humoral (cortisol, melatonin) or neural (autonomic nervous system fibers) outputs [19]. The SCN are entrained by environmental cues, mainly photic stimuli transmitted by the retino-hypothalamic tract and signaling environmental light-darkness alternation due to Earth’s rotation on its axis to tissues, organs, and organ systems [19].

Biological rhythmicity is generated at the molecular level by way of a group of intertwining genes with their encoded proteins, generating a transcriptional-translational feedback loop (TTFL) revolving with a frequency of 1 cycle in 24 ± 4 h [19]. The TTFL is operated by an activation branch, hard-wired by the Period-Arnt-Single-minded and basic helix-loop-helix (PAS-bHLH) transcription activators CLOCK (circadian locomotor output cycles kaput), and its paralog NPAS2 (neuronal PAS domain protein 2), and BMAL1-2/ARNTL-2 (brain and muscle aryl-hydrocarbon receptor nuclear translocator-like/aryl-hydrocarbon receptor nuclear translocator-like), which heterodimerize and rhythmically bind to E-box (5′-CACGTG-3′) cis-regulatory enhancer sequences of Period (Per1–3) and Cryptochrome (Cry 1–2) genes driving waves of epigenetic modification to promote gene transcription [20,21]. These genes encode PER and CRY proteins, responsible for the inhibitory branch of the feedback loop, accumulate and heterodimerize in the cytoplasm, translocate back to the nucleus, and impede CLOCK/BMAL1-2 transcriptional activity [20,21]. The circadian proteins go through various types of post-translational modifications (PTMs). The key regulators of the clock machinery, the serine/threonine protein kinases casein kinase (CK) 1 δ/ε and CK2, encoded by CSNK1D, CSNK1E, and CSNK2 genes, bind to and phosphorylate multiple circadian substrates [20,21].

Robustness and amplitude of the core TTFL is increased by the nuclear receptors (NRs) REV-ERBs and RORs, whose expression is rhythmically driven by CLOCK/BMAL1 heterodimer. In turn, REV-ERBs and RORs regulate BMAL1 transcription in competition with binding specific ROR response elements (RORE) in BMAL1 promoter and eliciting transcription inhibition and activation, respectively [20,21]. CLOCK/BMAL1 heterodimers bind E-boxes in the second intron of the PPARA gene and activate transcription of PPARA [22], which upon ligand binding, assists BMAL1 expression through a PPARα response element located in the BMAL1 promoter [23]. Furthermore, CLOCK/BMAL1 heterodimers drive the rhythmic expression of first order clock-controlled genes encoding proline and acidic amino acid-rich domain basic leucine zipper (PAR-bZIP) transcription factors, which control the rhythmic expression of thousands of downstream genes [24]. These PAR-bZIP transcription factors comprise DBP (albumin gene D-site binding protein), TEF (thyrotroph embryonic factor), and HLF (hepatic leukemia factor), and the bHLH transcription factors differentially expressed in chondrocytes protein (DEC)1 and DEC2. In addition, the interaction between the opposite oscillatory phase of DBP and REV-ERBs with RORs on RORE at the promoter of Nfil3/E4bp4 gene regulates the rhythmic expression of the nuclear factor interleukin 3 regulated protein (NFIL3, also known as adenoviral E4 protein-binding protein, E4BP4), which plays a key role in immune competent cell development and commitment [24,25].

## 3. The Role of Biological Clock Derangement in the Ageing Process

During the ageing process, progressive remodeling and degeneration of tissues also occur at the level of the cellular elements of brain structures. At the level of the SCN, there is a progressive reduction in the number of neurons expressing vasoactive intestinal peptide and arginine-vasopressin, with weakening of the connectivity in the functional network that ensures the robustness of the oscillation of neuronal and astrocytic cells. With the process of neuronal degeneration associated with aging, the network inside the SCN loses its links and progressively disintegrates, with a reduction in the amplitude of the oscillatory signal and a tendency to shorten the period of oscillation [26]. An important element affected by the ageing process is the pineal gland, which is part of the circadian timing system and whose secretion of melatonin decreases during ageing [27]. Another factor is cortisol, whose secretion by the adrenal glands is not reduced in amplitude but changes in its oscillatory pattern during aging. These alterations determine progressive derangement in the context of the circadian clock circuitry, with alteration of the harmonization of cellular processes and tissue functions in various organs and organ systems [28].

Senescent cells accumulate with aging, modulate their microenvironment through a particular secretory pattern (senescence associated secretory phenotype, SASP), and produce molecules with pro-inflammatory, proapoptotic, and pro-fibrotic activity, such as growth factors, cytokines/chemokines, and extracellular proteases, which not only have an autocrine effect, but also act on neighboring cells (paracrine effect). This bioactive secretome can impact the cell fate by triggering a senescence program and prompting cell-cycle arrest, preventing transmission of DNA damage to daughter cells, and thus preventing potential malignant transformation or, conversely, promoting proliferation, as induced by pro-inflammatory cytokines [29]. SASP is brought on by different signaling pathways, comprising the DNA damage response, stress kinases, inflammation and inflammasome activation, metabolic sensors/pathways, cell survival-associated transcription factors, autophagy, and chromatin remodeling. All these cell processes, primarily genetic modifications based on p16INK4a or p21CIP1/WAF1, are rhythmically driven by the molecular clockwork [30,31]. Lingering senescent cells, which harbor a failing biological clock and drive age-related disorders, pave the way for preventive/therapeutic strategies (senotherapy) that specifically aim to remove senescent cells with senolytics to curb ageing [32,33].

All the processes entailed in ageing are enhanced by the signaling pathways sustaining anabolism, such as growth hormone and insulin/insulin-like growth factor 1 signaling pathways, which activate PI3K-Akt-mTOR signaling pathway enhancing the aging-related processes [34]. Anti-ageing action is carried out by SIRT1-related signaling pathways, acting in part through PGC1alpha, and by AMPK, an important nutrient sensor. AMPK plays an antagonistic role with respect to mTOR signaling and directly modulates the molecular clockwork through phosphorylation of cryptochorme proteins, tagging them for proteasomal degradation [35,36]. Another important player in ageing is mitochondrial dysfunction. Aged and dysfunctional mitochondria must be destroyed and then renewed with processes of mitochondrial biogenesis, for which PGC1alfa and PGC1beta are fundamental. The biological clock is involved in mitochondrial reactive oxygen species (ROS) production and detoxification through the control of nutrient flux, uncoupling mechanism, redox regulation, antioxidant defense, and mitochondrial dynamics. An important mechanism is the excess of oxidizing radicals and decrease in antioxidant mechanisms [37]. The biological clock controls the fundamental processes that counteract damage from oxidizing radicals. Additionally, the role played by the hormone secreted by the pineal gland, melatonin, one of the most powerful molecules with an antioxidant effect is significant [38]. The derangement of the circadian clock circuitry causes a gradual decline in the production of antioxidant molecules in the face of a continuous increase in oxidative damage, as seen in the mouse model with loss of functionality of BMAL1, characterized by increased oxidative stress, impaired expression of several redox defense genes, increased neuronal susceptibility to oxidative damage, synaptic damage, and increased gliosis [39]. When the biological clock is altered, this results in a reduction in life span [40]. Gene mutations, especially in the core circadian genes, have an important impact on the duration of life. In experimental animal models, the knockout of BMAL1 gene results in accelerated ageing processes, increased oxidative damage, reduced body weight, sarcopenia, and altered leukocyte formula [34]. On the other hand, silencing of CLOCK results in reduced lifespan and increased incidence of cataracts [41].

## 4. The Biological Clock and the Innate Immune System

Inflammation, an adaptive host response also involved in tissue repair, alerts the immune system to sites of infection and tissue damage. Under normal conditions, at the molecular level, it is crucial to rigorously control the expression of genes enriching the signaling pathways that manage inflammatory responses by maintaining repression when external or internal stimuli are absent, and promptly activating their expression in the case of infection or tissue injury. The transcriptional control of a large part of these genes relies on signal-dependent transcription factors comprising members of the NF-κB, activator protein 1 (AP-1, a heterodimer composed of c-Fos/c-Jun family members), and interferon regulatory factor (IRF) families of transcription factors. After activation, NF-κB, AP-1, and IRF elicit the expression of numerous genes that turn on and boost inflammatory processes and support the progression of acquired immunity in different immune competent and inflammatory cells. Many receptor systems for molecules derived from pathogens (PAMPs), endogenous damage-associated molecular patterns (DAMPs), and receptors for cell-derived inducers of inflammation, such as IL1β, TNFα, interferons, can prompt the expression of these transcription factors [42].

The key of the circadian pathways in managing innate immune response was recognized by studying animal models with molecular clockwork changes. A pathophysiological connection between disruption of the molecular clockwork and increased susceptibility to chronic inflammatory diseases was suggested by studies performed in fibroblasts with double knock-out of cryptochrome genes (Cry1^−/−^Cry2^−/−^). These studies showed that in a cell-autonomous manner, dual silencing of the cryptochrome (CRY1 and CRY2) proteins remove the inhibitory effect on cyclic adenosine monophosphate (cAMP) production, increase intracellular cAMP levels, and enhance protein kinase A (PKA) signaling, with augmented phosphorylation of p65 at S276 residue and NF-κB signaling activation leading to unremitting increase in the proinflammatory cytokines IL-6 and TNF-α, and considerably augmented expression of inducible nitric oxide (NO) synthase (iNOS) [43]. On the other hand, homozygous Clock mutant mice showed reduced expression of immune-related genes and dampened oscillations of leukocyte number in the peripheral blood [6].

In Per2^−/−^ mice, serum levels of the proinflammatory cytokines interferon IL-1β and (IFN)-γ were significantly reduced after lipopolysaccharide (LPS) challenge, while TNFα, IL-6, and IL-10 production was preserved. Per2^−/−^ mice were more resilient to LPS-induced endotoxic shock with respect to wild-type mice, proposing mPer2 as an essential regulator of natural killer (NK) cell function [44]. Macrophages are key controllers of innate immunity and are responsible for time-based gating of proinflammatory cytokine secretion and systemic immune responses. Mouse macrophages enclose autonomous molecular clockworks driving 24-h rhythmicity in more than 8% of the transcriptome, controlling numerous essential regulators of pathogen recognition and secretion of cytokines, including TNF-alpha and IL-6 upon challenge with bacterial endotoxin at diverse daily intervals [16].

Studies performed on mutant mice and human macrophages showed that REV-ERBα blocks innate immune response to endotoxin driving genes implicated in innate immunity. This circadian gating on inflammatory pathways is lost in REV-ERBα^−/−^ mice [45]. TNF-α interferes with the functioning of the molecular clockwork, mainly thwarting the expression of PER3, DBP, TEF, and HLF, possibly triggering the so-called ‘‘inflammatory clock gene response’’ responsible for weakness in innate immunity activation and inflammatory diseases [46,47]. Innate immune response includes the acute phase response (APR), consisting of prompt gene expression reprogramming and metabolism adjustments upon inflammatory cytokine secretion and acute phase protein (APP) production in the liver, with increased APP levels characteristic of metabolic disorders. APR, along with lipid sensing nuclear receptors (NR) and signal-dependent activation of proinflammatory transcription factors, such as NF-kB, signal transducers, and activators of transcription (STATs) and AP-1 family members backs the interplay among metabolic and inflammatory pathways, sustaining the so-called “metaflammation” process and upholding atherogenesis [48]. Numerous NR involved in metabolic pathways, such as liver X receptor (LXR, binding oxysterols), peroxisome proliferator-activated receptor (PPAR, binding fatty acids), and farnesoid X receptor (FXR, binding bile acids) transcriptionally interfere with the pro-inflammatory signal-dependent initiating transcription factors NF-kB [homo- or heterodimer composed of p50 and p65 (RelA)], signal transducers, and activators of transcription (STATs) and AP-1 family members [49]. Upon ligand binding, these NR suppress inflammatory gene expression with a process called transrepression, which consists in NR–proinflammatory transcription factor complexes exclusion from genomic binding sites [49].

Close interactions among NR and the molecular clockwork influence its entrainment in diverse tissues: PER2 inhibits PPARγ expression and prevents its recruitment to promoters of target genes. Conversely, PPARγ activates BMAL1 transcription. There is a mutual activation of BMAL1 expression by PPARα and PPARα expression by BMAL1, while CLOCK cooperatively activates the 24-h rhythmic expression of PPARα [50]. During the inflammatory response, PPARγ expression can be activated by IL-4 and other immunoregulatory molecules, while it is suppressed by IFN-γ and LPS [51]. In addition, REV-ERBα increases the expression of IL-6 and cyclooxygenase-2 in primary human macrophages in blood vessel wall [52]. REV-ERBα expression is prompted by ligands binding to LXRs, which bind to a specific response element in the human Rev-erba promoter and regulate cholesterol turn-over in macrophages and their role in inflammation and immune response. Moreover, LXRα induces transcriptional expression of TLR4, while REV-ERBα binds as a monomer to a RE overlying the LXR RE in the TLR4 promoter and rhythmically inhibits LXR transactivation of TLR4 expression [53]. Furthermore, PER2 drives the rhythmic expression of the TLR9, which recognizes deoxyribonucleic acid (DNA) leading to circadian fluctuation of cellular activation and cytokine production influencing the immune response [54].

## 5. The Biological Clock and the Adaptive Immune System

The adaptive immune system operates clear-cut recognition of non-self-elements through the discriminatory expansion of cells primed to target definite antigens and implement immunological memory. Cells crucial for adaptive immune responses are T and B lymphocytes in cooperation with dendritic cells (DCs), which intercept non-self-elements and connect the innate and adaptive responses. All cellular components involved in adaptive immunity are equipped with biological clocks that rhythmically regulate their specific functions [55].

The major protagonists in adaptive immune response are CD4^+^ T cells (effector or helper T cells) which secrete cytokines and chemokines and assist in antibody production. The differentiation of naïve CD4^+^ T helper cells (Th) through Th1, Th2, Th17, and induced regulatory T (iTreg) cell lines is managed by the cooperation of a signal transduction web and a transcription factors set (STAT, GATA-3, NF-AT, NF-kB, and AP-1) whose expression is rhythmically driven by the molecular clockwork [56,57]. The transcription factors T-bet, STAT 4, eomesodermin, Hlx, and Runx3 are involved in the differentiation of Th1 cells, which secrete IFNγ, lymphotoxin α (LTα), and IL-2 and mediate immune responses against intracellular pathogens. The transcription factors STAT6, GATA-3, NF-AT, NF-kB, c-Maf, and AP-1 are involved in the differentiation of Th2 cells, which secrete IL-4, IL-5, IL-9, IL-10, IL-13, IL-25, and amphiregulin. Moreover, Th2 cells defense the host against extracellular parasites, including helminths. Furthermore, they are important in the induction and persistence of asthma and other allergic diseases [58].

Th cell differentiation is a specific and dynamic process accompanied by changes in expression patterns of thousands of genes at different stages. Once initiated, T cells develop several mechanisms to reinforce this program until epigenetic modifications are established in certain gene loci that lead to the acquisition of a stable and specific phenotype. The transcription factors DEC1 and DEC2 oscillate with 24-h periodicity in peripheral blood mononuclear cells and determine Th cells fate and function [59]. Faults in T lymphocyte activation and failed turn-over of activated T and B cells hallmark DEC1-deficient mice. On the other hand, DEC2 levels induced by IL-4 signaling gradually increase throughout Th2 cells differentiation upon inducing stimuli and mainly regulates IL-2Rα expression with upregulation of CD25 expression elicited by DEC2 upon STAT6 activation and IL-2 signaling [59]. An additional transcription factor greatly expressed in Th2 cells and in natural killer (NK) cell and NKT cells is NFIL3/E4BP4, which elicits transcription of genes regulating cytokine secretion and effector function [60,61,62].

PPARs play a key role in adaptive immunity. PPARγ activation in CD4+ T cells suppresses Th17 differentiation, impedes IL-4 production, and facilitates inhibition of IL-2 secretion by the T cells [63]. Unliganded PPARα negatively regulates the T-box transcription factor T-bet, a master driver of genetic programs in both the innate and adaptive immune systems, that specifies the Th1 lineage by inhibiting different T-cell fates. T-bet expression is induced by cytokines, such as IFN-γ, IL-12, IL-15, and IL-21 through the respective cytokine receptors. T-bet expression promotes downstream JAK/STATs or PI3K-AKT-mTORC1 signaling pathways by inhibiting p38 mitogen activated protein (MAP) kinase phosphorylation upon T cell activation [64]. The NR RORγt and RORα, along with STAT3, and Interferon regulatory factor–4 (IRF4), play a part in the differentiation process and function of Th17 cells, which secrete IL-17a, IL-17f, IL-21, and IL-22 and manage immune responses against extracellular bacteria and fungi [65,66,67]. Conversely, REV-ERBα negatively regulates Th17 cell development by competing with RORγt and driving the expression of NFIL3, which directly binds and represses the RORγt promoter. This results in modulation of Th17 signature genes [68,69].

RORα is also important for the development of the nuocytes, cytokine-secreting cells involved in type 2 immune responses that share developmental lineage with natural helper cells [70]. Alternatively, RORα is highly expressed in IgA (+) memory B cells and BMAL1 downregulation decreases B-cell development, determining dynamic transcriptional regulation of class-specific memory B cells [71]. In the absence of proinflammatory cytokines, TGF-β with FOXP3 and STAT5 drives differentiation of naïve CD4 T cells into induced regulatory T (iTreg) cells, which are essential in preserving self-tolerance in addition to regulating immune responses [72]. Aryl hydrocarbon receptor (AhR), a transcription factor of the bHLH/PAS family, crosstalks with the circadian clock circuitry and rhythmically mediates the biochemical and toxic effects of environmental xenobiotics and endogenous reactive by-products of normal metabolism through a specific receptor complex. Its activation promotes epigenetic changes modulating the differentiation of Foxp3 (+) Tregs and Th17 cells [73,74,75].

## 6. Ageing, Inflammation, and the Immune Response

Natural ageing is an unremitting and unrelenting process that commences in initial adulthood and is sustained by structural and functional changes at the cellular, tissue, and organ level. These changes are cumulative, progressive, intrinsic, and deleterious, and are associated with stress tolerance impairment and frailty, morbidity, and mortality in older adults. Other hallmarks of ageing are genomic instability, telomere attrition, mitochondrial dysfunction, autophagy insufficiency, cellular senescence and immunosenescence, chronic inflammation and inflammasome activation [4]. Pattern recognition receptors (PRRs) host sensor proteins largely expressed by innate immune effector cells to identify PAMPs and/or DAMPs. PAMPs are small molecules with conserved motifs connected with LPS from Gram-negative bacteria, while DAMPs are endogenous constituents discharged upon cell stress or death [76]. Based on protein domain homology, PRRs can be categorized as: Toll-like receptors (TLRs), retinoic acid-inducible gene I (RIG-I)-like receptor (RLR), nucleotide-binding oligomerization domain (NOD)-like receptor (NLR), C-type lectin receptors (CLRs), and absent in melanoma-2 (AIM2)-like receptors (ALRs) [77,78]. TLRs are mainly positioned intracellularly and on the cell membrane, while RLRs and NLRs are found in the cytoplasm [77]. Among the human TLR family, TLR3, TLR7, and TLR8 are in the endosomal compartment as endosomal RNA sensors [77]. Upon recognition of DAMPs, TLRs recruit intracytoplasmic toll-interleukin-1 receptor (TIR) domain-containing adaptor proteins, such as MyD88 and TRIF to initiate intracellular signal transduction and increase the secretion of proinflammatory cytokines, chemokines, and type I interferons (IFN) by immune cells [79,80,81].

Inflammasomes are intracellular multiprotein complexes located in the cytoplasm and composed of NLR protein 3 (NLRP3), adaptor apoptosis-associated speck-like (ASC) protein, and procaspasce-1. Upon activation, inflammasome formation prompts caspase-1 expression and upholds discharge of proinflammatory cytokines, IL-1β and IL-18 [82,83]. Assemblage of the NLRP3 inflammasome requires two components: The priming phase and the activation phase. Priming is necessary for NLRP3-mediated gene expression and encompasses post-translational modifications to organize precise inflammasome assembly. Upon inflammasome activation, NLRP3 polymerizes to ASC and recruits procaspase-1 to cleave pro-IL-1β into IL-1β combined with pro-IL-18 [82,83]. The NLRP3 inflammasome represents an essential element of the innate immune system through caspase-1 activation and proinflammatory cytokines IL-1β/IL-18 secretion in response to cellular damage [82,83].

As previously mentioned, the chronic, asymptomatic, low-grade inflammation occurring in the absence of infection during ageing is called “inflammaging” (alias inflammaging, inflammageing) [84]. Inflammaging has harmful effects on health and contributes to biological ageing and the development of age-related chronic diseases, such as atherosclerosis, chronic kidney disease, cardiovascular disease, adult diabetes, and Alzheimer’s disease. The specific causes of inflammaging remain largely unknown, but inflammation may be caused by: (i) Release by damaged cells and accumulation of altered molecules (microRNA, mitochondrial DNA or histones), which are recognized by the cells of the immune system, resulting in the activation and development of inflammation; (ii) increase in the number of senescent cells that release pro-inflammatory molecules into the blood; (iii) chronic stress conditions; (iv) derangement of autophagic processes; (v) alteration of the intestinal microbiota; (vi) immunosenescence, defining the steady waning of immune system function during ageing [85]. Autophagy and microbiome homeostasis are rhythmically regulated by the biological clock and their derangement cause several pathological processes underlying inflammatory, metabolic, degenerative, and neoplastic diseases. An in-depth discussion of the involvement of these processes in pathological conditions and in age-related diseases goes beyond the boundaries of this review; therefore, we refer to exhaustive articles already present in the international scientific literature [86,87,88,89,90,91,92,93].

Innate and adaptive immune response, including humoral and cellular immunity, decays during ageing. Increasing evidence shows that ageing significantly affects all cell compartments of the innate immune system. Numerous neutrophilic functions, for instance, phagocytic capacity, ROS production, and intracellular killing ability, are impaired in the elderly. Similarly, macrophage functions, including phagocytic activity, cytokine and chemokine secretion, antigen presentation, infiltration and wound repair and antibacterial proficiency decline with ageing. Age-related reduction in mast cell and eosinophil and alterations of functional properties have been demonstrated. Conversely, data regarding the influence of ageing on natural killer (NK) and natural killer T (NKT) cells numbers and functions are less evident [94].

Ageing-related changes in adaptive immunity include considerable reduction in the number of naïve lymphocytes formed in the bone marrow and thymus, in addition to the accumulation of functionally incompetent atypical B lymphocytes, named age-associated B cells (ABCs) [95]. The assortment of immune receptor repertoires is seriously restricted in aged B lymphocytes. Moreover, the helper function of naïve CD4^+^ T cells in B cells antibody production decreases during ageing [96,97]. Similarly, ageing disturbs the B lymphocyte stimulator (BLyS) cytokine family, an essential ligand class for B cell survival and maturation. ABCs exclusively react to TLR7 and TLR9 stimuli rather than B cell antigen receptor stimuli. Consequently, augmented ABCs are inclined to yield low affinity antibodies through the decreased aptitude of antigen recognition sites to identify antigens and activate inflammatory processes [96,97]. Furthermore, the number of naïve T cells that enable antigen presenting cells to communicate with antigen-specific CD8^+^ T cells is decreased during ageing [98,99].

**Figure 1:**
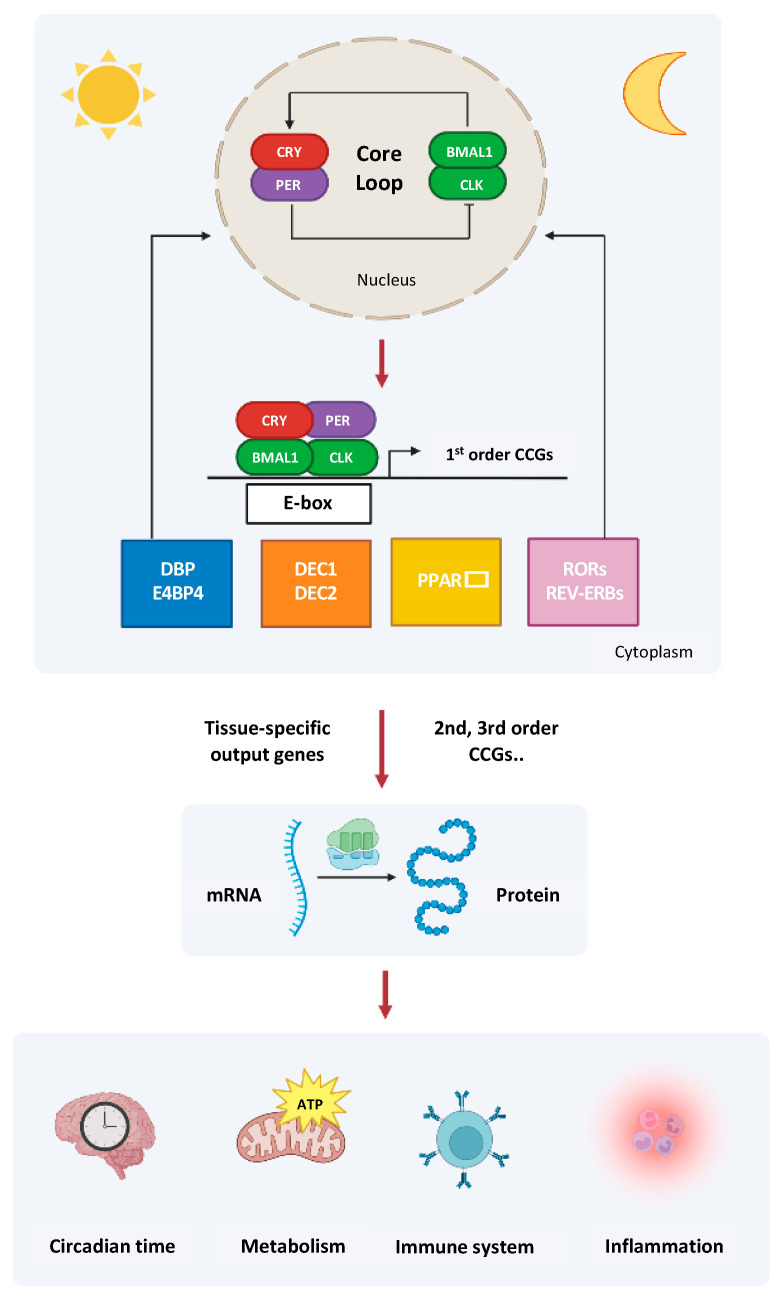
Schematic representation of the molecular clockwork driving the expression of thousands of downstream genes through transcription factors encoded by first order clock-controlled genes (CCGs). The second and third order specific output genes manage cell processes and tissue functions crucial for body homeostasis along with chronotype and taking part in ageing-related derangements.

A schematic representation of the molecular clockwork driving the expression of downstream genes is shown in Figure 1.

## 7. Endoplasmic Reticulum Stress and UPR during Ageing

Synthesis and folding of membrane and secretory proteins, lipids, and sterols, as well as free calcium storage take place in the ER [100]. Folding is the biophysical process through which the proteins arrange in their precise, three-dimensional outlines necessary for their specific biological function. Ageing is associated with changes in the expression of ER chaperones and folding enzymes, leading to the impairment of proteostasis, and accumulation of unfolded or misfolded proteins that build up in the ER causing ER stress and triggering UPR [100,101]. ER stress reestablishes homeostasis by prompting downstream gene transcription, together with ER overload response (EOR), sterol cascade reaction (SCR), and UPR. EOR is triggered by undue accumulation of correctly folded proteins in the ER and, in addition, prompts various signaling pathways. EOR includes Ca^2+^ release from the ER, ROS production, and prompting of nuclear factor kappa B (NF-κB)-dependent signaling pathway [102]. In conditions of cellular stress, EOR intervenes to re-establish in the ER proper efficiency in managing protein synthesis and folding, oxidative equilibrium, and Ca^2+^ flux [103]. SCR is started by cholesterol depletion, causing cleavage of the membrane-bound transcription factor sterol-regulated binding protein (SREBP), which prompts the transcription of genes controlling cholesterol absorption and biosynthesis as well as fatty acid synthesis and absorption [104]. During ageing, numerous factors, such as altered redox or Ca^2+^ homeostasis and protein glycosylation, could cause ER stress, disrupting correct protein folding and leading to an accumulation of misfolded proteins. Augmented ROS levels elicit pro-inflammatory signals with NF-κB activation in addition to NLRP3 inflammasome triggering and interleukin-1β secretion, hastening neutrophil recruitment, as well as endothelial adhesion molecules and cytokines/chemokines induction, T-helper cells activation, and B cells proliferation [105,106].

Protein quality control in the ER and activation of ER-transmembrane signaling molecules are managed by chaperone proteins, such as glucose-regulated protein 78 (GRP78) [107,108]. GRP78 interacts with the proximal sensors inositol-requiring transmembrane kinase/endoribonuclease 1α (IRE1α), protein kinase R-like endoplasmic reticulum kinase (PERK), and activating transcription factor 6 (ATF6) [109,110,111]. ER stress activates IRE1α, which oligomerizes and autophosphorylates through a kinase domain and upon activation splices and cleaves XBP1 mRNA to yield truncated XBP1 mRNA, encoding XBP1. Activated IRE1α mediates the splicing of an intron from the mRNA of XBP1, triggering a frameshift throughout translation and introducing a new carboxyl domain in the XBP1 protein, which was found to be a functional transcription factor [112]. This transcription factor begins the expression of genes managing synthesis of proteins essential for ER-related molecular chaperoning, protein folding, and degradation and adipogenesis [109,110,111].

Another UPR pathway is PERK, a protein serine/threonine kinase that oligomerizes and autophosphorylates on the kinase domain following ER stress and, in turn, phosphorylates eIF2α to hinder protein translation. Activated PERK enhances ATF4 transcription and translation to increase the level of proteins involved in vital cellular processes, including autophagy, amino acid turnover, protein secretion, redox equilibrium, and apoptosis. Furthermore, ATF4 binds a conserved site in the promoter of the PPP1R15A (GADD34) gene guiding a negative regulatory feedback loop that controls protein translation in response to ER stress [113]. ATF6 is an ER transmembrane protein working as ER-stress sensor/transducer and transcription factor that in cooperation with XBP1s binds cis-acting ER stress response elements (ERSE) and elicits the expression of genes encoding ER chaperones involved in UPR, protein folding, ER-associated protein degradation (ERAD), and mitochondrial biogenesis [109,110,111].

The biological clock drives an ancillary rhythmic activation with 12-h periodicity of the IRE1α pathway in the ER coordinating rhythmic expression of several ER-localized enzymes and UPR constituents involved in hepatic metabolism [114]. The intricate interplay between the circadian pathway and the UPR is operated by several components among which the microRNA miR-211 is crucial. ER stress impedes the transcription of core clock and clock-controlled genes via an ATF4-dependent mechanism [115,116,117].

## 8. UPR Mediators and Inflammation

IRE1α prompts inflammation via numerous mechanisms, especially through the regulation of inflammatory cytokine production and cellular signaling pathways, mainly through the kinase and endoribonuclease (RNase) activity of its C-terminal cytoplasmic region [118,119]. On the other hand, TLRs, particularly TLR2 and TLR4, can trigger IRE1α in mouse macrophages following stimulation by invading pathogens or endogenous signals, such as damaged cells [120]. After oligomerization, IRE1α RNase cleaves specific mRNAs through a definite process called regulated IRE1-dependent decay (RIDD) [121]. Moreover, IRE1α RNase prompts TNF-α production in macrophages through direct binding to its promoter/enhancer region [122]. Then, the activated IRE1α-TNF receptor-associated factor 2 (TRAF2) complex triggers JNK-AP1 and NF-κB and upholds IL-6 and TNFα production [122]. Furthermore, IRE1α-mediated activation of GSK-3β prevents XBP-1 splicing and transcription of IL-1β and amplifies inflammation [123], confirming that the IRE1 pathway is essential for unrelenting production of pro-inflammatory cytokines.

A pathway essential for inflammatory gene expression in the context of the ER stress-induced inflammatory response is represented by PERK-dependent phosphorylation of eIF2α with translation blockade. PERK-eIF2α-prompted translational block upholds IL-6, MCP-1, and CCL20 transcription independently of ATF4. PERK prompts NF-κB in a NOD1-dependent manner and triggers the JAK1/STAT3 pathway to uphold IL-6 production. Furthermore, the PERK/p38/ERK axis enhances IL-6 and IL-8 secretion [124,125]. The ATF6 family comprises seven members: The ATF6 paralogs (ATF6α and ATF6β) and five CREB3 proteins. ER stress and its specific signaling pathway mediated by ATF6 operates a crucial intracellular mechanism in regulating innate immune cells in vitro and innate immunity in vivo [126].

## 9. Ageing and NLRP3 Inflammasome Formation

The NLRP3 inflammasome is a cytoplasmic multiprotein complex consisting of the intracellular innate immune receptor NLRP3, the adaptor protein ASC (apoptosis-associated speck-like protein), and the protease caspase 1. NLRP3 inflammasome is initiated in response to extra- and intracellular danger signals, including mitochondrial oxidative stress. Upon activation, the inflammasome triggers an inflammatory response through the caspase-1-dependent activation of the cytokines IL-1β and IL18. The NLRP3 inflammasome recognizes PAMPs or DAMPs and triggers recruitment of the inflammatory protease caspase-1 [127], which modulates the inflammatory response through IL-1β and IL-18 precursors cleavage into active forms [128]. The NLRP3 inflammasome can be activated by various exogenous and endogenous stimuli, including pathogens, LPS, oxidized low-density lipoproteins, lysosomal damage, potassium leakage, ROS production and oxidative stress, Ca^2+^ ion mobilization and intracellular gradient [128,129]. NLRP3 inflammasome activation may also depend on ER stress as IRE1α activation enhances mitochondrial ROS levels supporting NLRP3 and mitochondria interplay.

Furthermore, ER stress triggers the inflammasome via NLRP3-caspase-2 driven mitochondrial damage and promotes inflammation, embracing cellular stress and innate immunity [130]. Similarly, IRE1α worsens ROS production and triggers the NLRP3 inflammasome to stimulate IL-1β maturation in long-lived, donor-reactive memory B cells (BMEMs) throughout ER stress [131,132]. NLRP3 inflammasome activation is enhanced by mitochondrial ROS production upon Ca^2+^ release from the ER during stress. Moreover, NLRP3 inflammasome activity is regulated through ROS and mitochondria-associated membrane (MAM) proteins “diaphony” [133]. NLRP3 expression and activation, as well as IL-1β and IL-18 secretion in various tissues and immune/inflammatory cells, particularly macrophages, is controlled by the biological clock, which drives 24-h rhythmic fluctuations of NLRP3 signaling.

Studies performed in peritoneal mouse macrophages in vivo showed that REV-ERBα transcriptionally inhibits expression of the NLRP3 inflammasome components, whose mRNA levels peak throughout the active phase, coinciding with the lowermost intracellular levels of REV-ERBα [134]. On the other hand, decreased Nlrp3 and Il1b mRNA levels and diminished ability to secrete IL-1β was shown in bone marrow-derived macrophages isolated from RORγ-null mice, and numerous presumed ROREs occupied by RORγ were discovered in the Nlrp3 and Il1b genes promoters [135].

## 10. The Circadian Epigenome and the Epigenetic Clock in Ageing

The functioning of the molecular clockwork and the rhythmic bursts of gene transcription depend on definite and cyclic chromatin changes operated by circadian chromatin remodelers and occurring on a genome-wide scale [136]. The transactivation capacity of the core protein CLOCK on E-box–containing promoters is linked, at least in part, to its histone acetyltransferase (HAT) activity on histone H3 at K9 and K14, with recruitment of other HATs, i.e., CREB binding protein (CBP), p300, P300/CBP-associated factor (PCAF), thus determining chromatin modifications that increase DNA accessibility and enable gene transcription [137]. HATs are counterpoised by a group of histone deacetylases (HDACs)-containing repressive complexes rhythmically recruited to chromatin, such as SIN3A–HDAC1 complex recruited by PER proteins, SIN3B–HDAC1/2 complex recruited by CRY1, and NCoR–HDAC3 complex recruited by the nuclear receptor REV-ERBα [137].

Among HDACs, a peculiar role is played by a class of seven enzymes, communally identified as sirtuins and implicated in many processes linked to metabolism, inflammation, and ageing. The nicotinamide adenine dinucleotide (NAD+)-dependent class III HDACs sirtuin (SIRT) 1 modulates the transcriptional activity of CLOCK:BMAL1 heterodimer in the nucleus [138]. SIRT6 plays a role in the rhythmic mitochondrial function driving acetylation/deacetylation cycles at the level of Complex I. Precisely, circadian oscillations in the activity of nicotinamide phosphoribosyltransferase, the limiting enzyme in the NAD+ salvage pathway encoded by the clock-controlled gene NAMPT, determine 24-h fluctuations of cellular NAD+ content, a crucial nutrient sensor. Moreover, they fuel the cyclical deacetylation of a single subunit of the respiratory chain Complex I and, ultimately, the rhythmic activity of mitochondrial respiration [139,140]. In addition to acetylation, other post-translational chromatin modifications have an impact on the ticking of the biological clock, such as H3K4me3 activation operated by members of the mixed-lineage leukemia (MLL) family histone methyltransferases (HMTs), and the H3K4 histone methyltransferase (HMT) MLL1, which induces cyclic trimethylation of H3K4 and recruitment of CLOCK:BMAL1 in chromatin at definite circadian gene promoters [141]. In addition, the clock-controlled histone-remodeling enzyme MLL3 epigenetically targets and modulates circadian transcription resulting in a whole-genome cycle of activating (H3K4me3) and inhibitory (H3K9me3) chromatin marks [142]. Similarly, the methyltransferase EZH2 determines the repressive mark H3K27me3 at the promoter of Per1 [143]. Moreover, the JumonjiC and ARID domain-containing histone lysine demethylase 1a (JARID1) act as an inhibitor of HDAC1, thus increasing CLOCK:BMAL1 transcriptional activity [144]. The flavin adenine dinucleotide (FAD) dependent demethylase LSD1 acts as circadian chromatin remodeler, controlled by PKCα-mediated 24-h rhythmic phosphorylation [145].

Another important level of epigenetic modification and gene transcription regulation is the transfer of a methyl group from donor S-adenyl-l-methionine (SAM) through a set of DNA-modifying enzymes with covalent addition and methylation at cytosine bases. This process is operated by a conserved and highly regulated family of DNA methyltransferases, among which the canonical DNMT enzymes are DNMT1, mainly involved in maintenance methylation, and DNMT3A and DNMT3B, principally operating de novo methylation. In eukaryotic genomes, common methylation marks occur as methylation of the carbon-5 of cytosine (5 mC), generally present on cytosines preceding a guanine nucleotide (also known as CpG dinucleotide sites) and occur on both DNA strands to maintain the post-replicative DNA methylation patterns [146]. Remarkably, experiments performed in animal models and human cells showed circadian oscillation of global DNA 5mC content and 24-h rhythmic changes in canonical DNMTs mRNA levels and enzymatic activity, suggesting a crucial role played by the biological clock in the control of maintenance and de novo DNA methylation [147,148,149].

Age-associated deregulation of the epigenome characterizes the ageing process, partly due to an alteration in the circadian clock circuitry [150]. Disruption of the biological clock has been associated with numerous diseases and age-related processes at the transcriptional, translational, and post-translational level, as well as with age-specific signatures at the genomic, genetic, and epigenetic levels [151,152]. In all species, tissues, cell types, and epigenetic traits change dynamically throughout life. Specifically, during the ageing process, DNA methylation patterns modify leading to genome-wide hypo-methylation and site-wide hyper-methylation. These epigenetic drifts are generally erratic and non-directional, limiting prediction of both DNA hypo-methylation and hyper-methylation, with different patterns of changes in the methylome among ageing individuals. Nonetheless, there is plausible evidence of ageing-associated differentially methylated regions, through consecutive groups of cytosine-phosphate-guanine (CpG) dinucleotides. These sites could show non-stochastic methylation changes in a constant direction over time and could be related to biological mechanisms strictly associated with the ageing process and longevity. They represent DNA methylation clocks, generally referred to as epigenetic clocks, and confer DNA methylation-based approximations of biological age (Horvath’s clock, Hannum’s clock, DNA PhenoAge, and DNA GrimAge), developed through joint exploitation of mathematical algorithms, mostly based on machine learning. The sets of CpGs are robustly correlated with age, but not convincingly with a decline in physical function. Indeed, additional studies are needed to elucidate whether epigenetic ageing renders the decline in muscle strength with ageing or whether epigenetic clocks simply measure the evolution of ageing [153,154,155,156].

## 11. “Circadian Medicine”: A New Anti-Ageing Therapy

“Circadian medicine” or “Chronotherapy” is a new model of anti-ageing intervention. It is based on current evidence that circadian rhythms are reduced and more likely to be altered with age, leading to metabolic disorders that reduce longevity [157,158,159]. An alteration of circadian rhythms can also cause a direct disruption of age-related pathways, that oscillate physiologically during the day [160,161]. In light of this evidence, it is hypothesized that there is an optimal time for the administration of drugs that can restore the correct target rhythms, and ultimately improve health and prolong lifespan.

There are many treatments for age-related diseases that are more effective when administered at specific times of day, particularly those for cardiovascular diseases (CVD). One of the most illustrative examples is aspirin, used as a secondary prevention of CVD in humans. A randomized crossover study showed that aspirin reduces blood clotting more effectively when taken at bedtime rather than in the morning [162]. These results were also confirmed by a placebo-controlled study conducted in healthy adults over the age of 65, in which it was observed that those who received aspirin at breakfast, rather than at dinner, experienced an increase in bleeding, CVD risk, and all-cause mortality [163]. Similarly, the efficacy of the statin simvastatin and antihypertensive drugs, such as the Ca^2+^ channel blocker nifedipine and angiotensin II receptor antagonists, is greater when taken before bedtime [164,165].

Other treatments for age-related diseases act directly on the activity of the internal clock. Polyamines represent a pharmacological intervention that mediates the crosstalk between the circadian clock, metabolic pathways, and lifespan. Indeed, these molecules regulate circadian periodicity via PER2/CRY1175 interactions leading to increased lifespan in mice [166]. Another molecule that enhances clock action is a natural flavonoid called nobiletin (NOB). This flavonoid has been found to reduce body weight gain without altering food intake, stimulate energy expenditure and circadian activity, improve glucose and insulin tolerance, decrease lipids, and improve mitochondrial respiration in mice [167,168]. These findings clearly demonstrated that maintaining a strong circadian organization in the body protects against metabolic perturbations.

Considering the promising evidence obtained to date, future human studies that consider tissue-specific circadian fluctuations in drug treatments to counter age-related diseases are needed. Providing guidance on when and how often treatments should be given is crucial to reduce drug resistance, side effects, and promote healthy ageing.

## 12. Conclusions

The processes that undermine ageing include immunosenescence, inflammation, and inflammasome formation in the context of ER stress and autophagy derangement. Innate and adaptive immune response is characterized by rhythms with diverse frequency ranges, generally with 24-h periodicity and with coincident or opposing phases in the levels of cellular effectors and humoral factors. Biological clocks ticking in inflammatory/immune cells drive fluctuations of leukocyte trafficking and turn-over, phagocytic and peroxidase activity, as well as cytokine/chemokine secretion and antibody production. The circadian clock circuitry and the immune system intermingle bidirectionally integrating environmental cues with the internal milieu. Signal-dependent transcription factors and nuclear receptors maneuver transcriptional circuits and gene signature determining inflammatory/immune cells’ function and fate through mutual communications linking the molecular clockwork to the rhythmic regulation of inflammatory pathways and immune response. Increasing evidence highlights the role played by changes in the circadian clock circuitry during ageing and the possible nutritional and pharmacological interventions aimed at limiting or delaying these alterations [169,170]. A deeper understanding of the molecular alterations occurring within the interactions between the circadian pathways, the inflammatory process, and the immune response, especially in the context of chronodisruption, would allow us to ascertain and therapeutically address some of the age-related pathophysiological mechanisms involved in inflammatory response and immune system dysregulation during ageing.

## Data Availability

The data presented in this study are available in the article.
